# Exploring faculty perspectives toward developing a planetary health curriculum for family medicine residents at the University of Toronto

**DOI:** 10.3389/fpubh.2025.1614872

**Published:** 2025-07-22

**Authors:** KitShan Lee, Elisabeth-Abigail Ramdawar, Samantha Green, Rachel Adilman, Azzra Mangalji

**Affiliations:** ^1^Department of Family and Community Medicine, University of Toronto, Toronto, ON, Canada; ^2^Toronto East Health Network – Michael Garron Hospital, Toronto, ON, Canada; ^3^The Wilson Centre, University Health Network, Toronto, ON, Canada; ^4^Institute of Health Policy, Management, and Evaluation (IHPME), Dalla Lana School of Public Health, University of Toronto, Toronto, ON, Canada; ^5^Unity Health – St. Michaels Hospital, Toronto, ON, Canada

**Keywords:** medical education, curriculum development, community health/public health, faculty development, planetary health, family medicine

## Abstract

**Background:**

Climate change is the greatest threat to human health of this century, yet limited formal curriculum exists within postgraduate family medicine (FM) programs across Canada. As outlined by The College of Family Physicians of Canada (CFPC) Guides for Improvement of Family Medicine Training (GIFT) report, learners have called for planetary health (including climate change) education and recommended a curriculum framework. This study aimed to understand University of Toronto Department of Family Medicine faculty attitudes around implementing a planetary health curriculum within the FM residency program.

**Methods:**

This study used a qualitative descriptive design. Thirty faculty members from various teaching, curriculum, and leadership positions were invited to participate in virtual semi-structured video interviews. Data was collected and analyzed using thematic analysis.

**Results:**

Thirteen interviews were conducted between May–September 2022. Participants perceived planetary health was relevant to FM, but most were unfamiliar with the term. Four overarching themes were developed from the data: (1) curriculum implementation, (2) curriculum development, (3) barriers, and (4) attitudes. Barriers to integrating PH learning objectives include a lack of faculty knowledge and skills, burnout, and an already saturated FM curriculum.

**Conclusion:**

To address the climate crisis, there is need for a planetary health curriculum, yet faculty have a limited understanding of this topic. This knowledge gap is one of multiple barriers to curriculum implementation this study identified. This study provides insight and suggestions for tools that may aid planetary health curriculum development and implementation.

## Introduction

The global community is reported to be facing a triple planetary crisis, encompassing the inter-related challenges of climate change, pollution, and biodiversity loss, all of which pose a significant threat to public health (1). Planetary health is defined by the Rockefeller Foundation-Lancet Commission as *‘the health of human civilization and the state of the natural systems on which it depends’* (2). It is a transdisciplinary field which not only focuses on human health impacts, but also calls for urgent action to combat the extensive degradation of our planet for human advancement (2). Although similar to One health and Eco Health, planetary health emphasizes the interconnected relationship between climate change, biodiversity loss, environmental degradation, and its profound impact on human health and well-being (3). Furthermore, planetary health calls for a comprehensive systemic response, integrating societal, political, and economic strategies (i.e., the involvement of stakeholders across disciplines and fields) to address global health challenges (3, 4). The World Health Organization (WHO) has declared climate change as the greatest health threat of this century and it is already affecting the health of Canadians (5, 6). Climate change is a threat multiplier, affecting human health both directly and indirectly (e.g., physical and mental health), undermining many of the social determinants for good health, creating new public health challenges (5, 7). Although climate change impacts everyone, vulnerable and marginalized groups, such as children, older adults, Indigenous communities, racialized populations, individuals with low income, and the politically marginalized, are disproportionally affected (5, 8). Moreover, if the healthcare sector were a country, it would be the fifth-largest emitter on the planet (9), accounting for 4.6% of Canada’s greenhouse gas emissions (10). As the foundation of medical care in Canada, family physicians (FPs) serve a crucial role in educating their patients and communities about emerging public health issues and risks in order to mitigate harm. Multiple calls to action have urged FPs to take stronger steps to address the climate crisis (11–18). Mitigating climate change is both an issue of social accountability for FPs, and an opportunity to counsel patients on the many health co-benefits of climate action (19).

As family medicine training programs have a distinct responsibility to prepare FPs to respond to and advocate for the health needs of society (20), calls to educators have been made to incorporate planetary health concepts into training programs (15–24). The 2020 Guide to Improving Family Medicine Training highlighted the need to incorporate planetary health concepts into FM residency training in Canada (22). However, prior studies have identified that a lack of faculty knowledge, bandwidth, and comfort are common barriers to curriculum development and implementation (23, 24). The purpose of this study was to explore the University of Toronto’s (UofT) Department of Family & Community Medicine (DFCM) faculty perspectives toward planetary health education within the FM residency program.

## Methods

This study used a qualitative descriptive design (25) and received approval from the UofT Research Ethics Board. Using a purposive sampling method (26), 30 DFCM faculty, consisting of clinician teachers, residency program directors, curriculum leads, and senior leadership, were invited to participate in semi-structured interviews. After written consent was retrieved, interviews were recorded, transcribed, and de-identified. Three research team members (EAR, RA, AM) performed independent thematic analysis (27–32). Two team members (KL and SG) served as triangulators to reduce intrinsic biases, prejudice, and perceptions (27–32). Using NVivo 11 and Microsoft Excel, themes and codes were logged, reviewed, and refined. Thematic saturation was believed to have been achieved based on repeated data patterns in themes and subthemes (33).

## Results

From May–September 2022, 13 DFCM faculty participated in semi-structured video interviews lasting approximately 45 to 60 min. All participants expressed the importance of planetary health in FM. Most indicated a lack of knowledge and skills to deliver a planetary health curriculum and noted the need for improved understanding of planetary health and teaching methods among faculty.

Four overarching themes and several subthemes developed (see Table 1).

1. ***Curriculum Development*** focused on a need for evidence-based curriculum, that is relevant to all parties, which incorporates social accountability, and faculty role modeling.
2. ***Curriculum Implementation*** including expert training to both undergraduate and postgraduate faculty, and culture change with the application of a planetary health lens to all aspects of clinical practice.
3. ***Attitudes and Perceptions*** were highlighted for resident and faculty buy-in, coupled with the need for support from local and general leadership.
4. ***Barriers*** included pervasive faculty burnout along with an already saturated curriculum, time constraints, and lack of resources including knowledge.

**Table 1:** Developed themes and sub-themes with quotations.

Subthemes	Quotations
Theme 1—Curricular development	
Clinically Relevant to: Institution Faculty Learners Patient Care	“I think that we, as medical providers, want to look at some of the evidence, and **we need to know this is the truth** before we will start believing it and acting it on it, so having some lectures, or like a curriculum that is responding to develop that, it would be great.” *Interview 6* “It makes sense that **now is the time that we are starting to understand our responsibility as medical teachers to understand and include this kind of stuff**, and the impact of climate change on our patients’ health, as well as advocate as healthcare professionals for the societal change that we need to combat it.” - *Interview 8* “**It takes some pretty deep blinders in 2022** to say that the segment of the healthcare workforce that is the foundation of the entire Canadian healthcare system - and provides the bulk of primary care as a planet is evolving the way it is - **should not be taught formally about climate change** […] The question is where and how and what, like what is it about climate change that we need to teach residents.” - *Interview 2* “…**we are the boots on the ground**…We are the first contact with patients in all facets of their healthcare, and we are probably the ones that are going to see first the impact, as well as potentially have the opportunity to mitigate those impacts before they even encounter specialist colleagues and hospital care.”- *Interview 8*
Social Accountability and Justice	“There is no question that **climate change**, and many other social determinants of health, **have greater impact on our patients’ health than a lot of what we can do in the office**…and there is no question that as family physicians we have a particular lens into that piece of it.” - *Interview 1*
Stakeholder Engagement	“I think there is a **willingness** at this point, **both at the learner level and at the teacher level**, and an understanding that this is really important and critical, that will hopefully help to kind of push this over the finish line and get this kind of stuff incorporated.” - *Interview 2*
Future Initiatives	“It would be great for the College of Family Physicians to say if you have never taken a planetary health course, you need to do this at least once in every five-year cycle, the same for indigenous health. I think if you are a Canadian family doctor, maybe you do not need to do this every year, but you need to do this at least once every five or ten years.” - *Interview 2*
Evidence Based Curriculum	“That is the homerun of any new curriculum, that we actually do it under a scholarship lens, and **we prove that it is beneficial**. So, that takes money, and usually a research grant. It takes time.” - *Interview 1*
Theme 2—Curriculum implementation	
Change in culture Buy in for sustainable practice Funded leadership positions	“**If we are all thinking, or most of us are thinking, that climate change is an important problem**, then it will **inform** how we talk and communicate, and then that will reinforce whatever we do in a formal curriculum.” - *Interview 1* “…you have to have **champions**, and you have to have **people in leadership positions who have knowledge of planetary health** in order to be able to provide that teaching to residents.” - *Interview 8*
Enabling curriculum delivery Expertise Faculty Education Methods of Integration	“I think having **the development of it led by people who actually work in the field** and at least take an interest in it…how do you apply this to your clinical practice on a day-to-day basis, because theory can be quite interesting, but I think the residents are very keyed into…**what is the clinical practice pearl.**” – *Interview 5* “It is not about having an academic half day on climate change; it is about **how does it impact every single thing we think about**, and we teach you, and get us into the habit of thinking about it in that way. […] I do not think it is as simple as popping an academic half day in there and you are done. **I think it has to be everywhere**.” - *Interview 8*
Theme 3—Attitudes and perceptions	
Professional Responsibilities	“How do we bring it in in a way that is tangible and meaningful to our learners, so they can meaningfully incorporate this into their future practice […] versus just it **being another thing that they learn about, feel overwhelmed by, and kind of do not know what their job is**.” - *Interview 1*
Personal Motivation	“You will always have your **passionate folks** who will listen to any lecture on it, and be like, ‘Oh, thank god they are teaching this’, but you are going to have a whole bunch who are like, ‘Okay, but when do we get to diabetes?’” - *Interview 1*
Medical Education	“I think it **belongs in curriculum all across, and not just family medicine residency.** […] If you are talking about respiratory health, really it is across the division of medicine and things like that. Is it appropriate for family medicine, absolutely. Is it only limited to family medicine, no.” - *Interview 7*
Stakeholder Roles and Power Dynamics	“I can say with absolute certainty that people do value the view of the Chair, [but]…**nothing moves in this department without the grassroots bottom-up initiative also**. Having said that, support from the top was important in Indigenous Health. **What I was referring to there was money**. We are paying for the San’yas training for all of the sites that cannot find those dollars locally and, yes, we have stated that it was a priority.” - *Interview 13*
Perceived relevance to core competencies	“The tricky part is **where does our training begin and end with this**, and how much of this can we take on, and should we be expecting our residents to take on as a part of their future practices.” - *Interview 1*
Perceived patient priorities	“I am least likely to do this with my patients who are poor and marginalized … these are already people who are having a very hard time often taking good care of themselves because society does not allow them to.” - *Interview 2*
Perceived lack of resident buy-in	“I think it is going to be really hard…most of our residents, not all, but **most of our residents are so focused on gaining** -- I hate to say it, but I am going to say **core clinical skills**.” - *Interview 1* I can see some residents where there **might not be buy-in**…. if we as individuals cannot necessarily see the direct and immediate impacts of climate change on our own health,… then they might not have buy-in to learn about it, or feel it is most relevant to their training. - *Interview* 9
Theme 4—Barriers	
Bandwidth and Burnout	“I feel like everybody is kind of at their max, and even **the prospect of taking on a whole new initiative right now feels a little bit overwhelming**.” - *Interview 8* “Never mind sustainable practices, like our practices are a mess for a whole bunch of reasons. We cannot get our printers to work half the time, which I appreciate is another sustainability issue. **We are just trying to keep our heads above water**.” - *Interview 1*
Change is Slow	“My experience has been that **it takes much longer for things like this to trickle into medical curricula**, and I think that has to do with just the many layers of things to go through before you can get something into curriculum.” - *Interview 10*
Competing priorities	*“*I think a lot [of residents] are still trying to get through their day of clinic and their projects. I think for most of them they are going to **shut down because they are not ready for it**. It’s not because it is not important, but I think we have to understand the number of things they are asked to learn in a very short period of time.” - *Interview 1*
Saturation of Content	“I will just quickly tell you -- **nothing has ever been taken out**. We never said, ‘Oh geez, you know, family doctors do not need to know this anymore, we will take it out.’ Okay? So, the in and out does not exist. It is piling on.” - *Interview 12* “**Almost everything belongs in the family medicine curriculum,** and we have this discussion many times a year with students especially interested in certain things, all very important things, and **we actually sometimes have to resist adding stuff to the curriculum because it is just too much**.” - *Interview 3*
Time Constraints	“One of our problems, our sites and core rotations are blocked out a couple of years in advance, and as a Program Director, if I ask the sites to say, ‘Listen, I need an hour with your residents sometimes between now and the end of the year,’ they are going to say, ‘**Where are we going to find that time?**” - *Interview 12*
Institutional Barriers Academic vs. Community sites Policies Resource Allocation for Curricular Change	“The first piece is **putting the leadership in place**; it is there. The second piece is actually **looking at the competencies** and the curriculum; is it already there? What are the changes to be made, and then can we execute them, and then going through the process of approval through the curriculum committee, and then approval to RPC, but the ultimate thing is - can you create the learning environment at all 15 of our sites that can fulfill the competency?” - *Interview 12*
Faculty Knowledge Gaps	**It is not obvious**, and I think that is the challenge that many of us experience. It is not just us providers or patients, but just as people. **We know that climate change exists, but I do not think we are seeing on an individual level that it is directly impacting our health**, even though I know what the impact of climate change on health can be. - *Interview 9* “One of our biggest problems is to remember curriculum is taught and assessed by faculty, and **when faculty do not know the curriculum**, or know the competencies within the curriculum, it is hard to teach it, and **it is hard to move it forward**.” - *Interview 12* This is not new and this is not new information that we have, but **it is kind of new in the way that we are thinking about it with its impact on healthcare**, and so when I think about our local expertise, for example, to have us as on the ground teachers for our residents, **role modeling how to incorporate** these considerations in terms of how we care for our patients, I do not have the expertise to really fully understand it. I think there will need to be faculty development in addition” - *Interview 8* “I feel like I do not know enough about it to be able to engage in it. As I said to you before, **I have not had any training in this, so how can I be engaged in that**?” - *Interview 7*

## Discussion

Despite multiple calls to integrate planetary health into FM education curricula, minimal planetary health content has been implemented in the UofT FM program. With new planetary health objectives being integrated into CanMEDS 2025 (21), and evidence that medical learners are interested in planetary health (22–24, 34–38), there is a need for curriculum development in post graduate medical education (21, 24).

**Figure 1:**
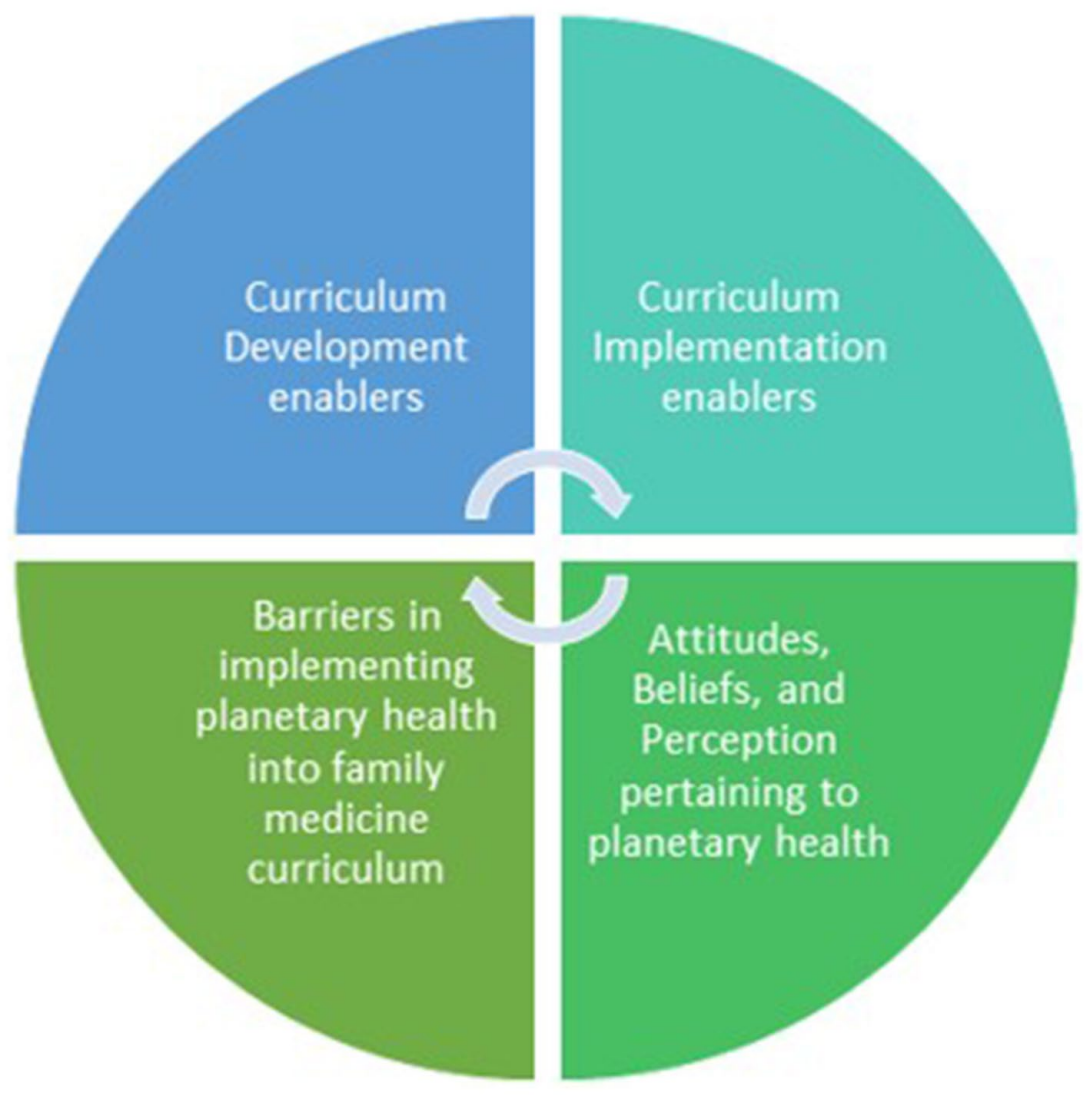
Faculty’s attitudes, beliefs, and perceptions may be indirect barriers in implementing planetary health into family medicine, which informs of potential enablers that would facilitate planetary health curriculum development and implementation at the DFCM.

Participants cited many barriers to implementing a planetary health curriculum, including personal (e.g., expertise, bandwidth, time), and institutional barriers (e.g., saturated curriculum, lack of expertise, lack of time in residency program), in keeping with prior studies (23). These findings align with Luo et al., who reported two significant barriers to integrating universal planetary health medical curricula in Canada: (1) limited curricular space for new content and (2) a lack of faculty expertise for teaching and role-modeling (35). Faculty attitudes and beliefs may also be indirect barriers to implementing planetary health content in FM residency programs. Understanding such challenges will help facilitate and enable planetary health curriculum development and implementation (Figure 1).

As most faculty expressed a lack of knowledge in planetary health concepts, using adaptive and transformational learning approaches would likely be best suited for the clinical challenges of planetary health. Adaptive experts are skilled at innovative problem solving in response to novel practice challenges and are prepared to remain curious and engaged in learning to meet the changing needs of their future practice (39). Transformative Learning is an educational theory that drives “dramatic, fundamental change in the way we see ourselves and the world in which we live” (40). The outcome of transformative learning is the development of a more open and inclusive worldview (41) that may lead to more lasting individual or systems change (42).

Practice-based learning and improvement has been proposed as a possible Transformative Learning method in health professional education (43). Through critical reflection on a disorienting dilemma, learners may improve their own clinical practice, recognize injustices in the health care system, and learn to intervene at the micro, meso, and macro levels (43).

To address faculty knowledge development while balancing burnout concerns, educational tools must be directed at faculty (44). Faculty development efforts must be offered in acceptable, interesting, and approachable ways (44). Moreover, as expressed by participants, addressing faculty development needs—especially in areas of support, expertise development, and confidence—is crucial for overcoming barriers to planetary health FM curriculum implementations. As such, the use of planetary health education leadership such as co-ordinators (4) or directors (45), may effectively mitigate these challenges. Additionally, blended learning, a combination of face-to-face and e-learning, has been found to be superior to traditional learning on knowledge outcomes (46).

We propose the following as methods for faculty development:

1. Brief 10-min presentations on select, clinically relevant planetary health topics to FM residency training sites for review at team/teachers’ meetings.
2. An asynchronous e-module on planetary health developed by local experts that is easily accessible by both faculty and residents Refer to Feldman et al. https://rise.articulate.com/share/DcYCoaIt75885kj403ISZbneuu3sFrbd#/ as an example (47).
3. Incorporation of planetary health teaching into existing curriculum, such as Quality Improvement (QI) programs, to provide both transformative and adaptive learning opportunities.
4. An asynchronous case-based learning module, which both residents and faculty could complete together. There is precedent for learner-led curriculum development and learner/faculty co-education in planetary health (48), and these ideas are likely applicable to residency education. This would be clinically meaningful and relevant and would enable faculty and residents to learn about planetary health together, with minimal local expertise. Using the module as a facilitator of interaction between learners and teachers, in a setting conducive to planetary health reflection, would provide benefits beyond educational needs.

To our knowledge, this is the first study investigating perceptions of FM faculty toward a planetary heath curriculum in Canada. As this study was conducted only at a single university, and given the challenges in implementing planetary health curricula, generalizability may be limited. However, findings from this study may be transferable and relevant to other medical disciplines and health professional training contexts.

## Conclusion

A planetary health curriculum in FM postgraduate training is critical to equipping future FPs with the necessary knowledge and skills to face the health impacts of climate change and other ecological crises. FM faculty development, provided in a way that is sensitive to burnout and time constraints, is required to teach planetary health objectives. Using adaptive and transformational learning techniques, faculty development can be done in a multi-modal way to improve efficiency and uptake. Incorporating a planetary health lens into existing curriculum would reduce time burden into a busy curriculum.

## Data Availability

The datasets presented in this article are not readily available due to confidentiality and is to be destroyed after 5 years. Requests to access the datasets should be directed to kitshan.lee@utoronto.ca.
